# UC-MSCs exhibit superior antifibrotic and anti-inflammatory effects compared to BM-MSCs in a bleomycin-induced idiopathic pulmonary fibrosis model

**DOI:** 10.1515/biol-2025-1293

**Published:** 2026-03-10

**Authors:** Yung-Lun Ni, Huan-Ting Shen, Chien-Ying Lee, Tai Ping Lee, Martin Sieber, Ching-Chi Tseng, Chang-Yao Thomas Tsao, Yu-Hsiang Kuan

**Affiliations:** Department of Pulmonary Medicine, Taichung Tzu Chi Hospital, Buddhist Tzu Chi Medical Foundation, 427 Taichung, Taiwan; Department of Pharmacology, 34899 School of Medicine, Chung Shan Medical University, 402 Taichung, Taiwan; Department of Pharmacy, Chung Shan Medical University Hospital, 402 Taichung, Taiwan; BIONET Therapeutics Corp., 114 Taipei, Taiwan; BIONET Corp., 114 Taipei, Taiwan; Department of Dermatology, The Wilshire Lab and Aesthetic Clinic, 518054 Shenzhen, China; Department of Dermatology, Shiso Municipal Hospital, 671-2576 Hyogo, Japan; Division of Chest, Department of Internal Medicine, Chung Shan Medical University Hospital, 402 Taichung, Taiwan

**Keywords:** idiopathic pulmonary fibrosis, umbilical cord-derived mesenchymal stem cells, bone marrow-derived mesenchymal stem cells, bleomycin

## Abstract

Idiopathic pulmonary fibrosis (IPF) is a serious lung disease characterized by excessive tissue buildup and reduced lung function, with few effective treatment options available. This study investigates the effectiveness of umbilical cord-derived mesenchymal stem cells (UC-MSCs) compared to bone marrow-derived mesenchymal stem cells (BM-MSCs) in a mouse model of bleomycin-induced IPF. Male BALB/c mice were divided into four groups: control, bleomycin-induced pulmonary fibrosis, UC-MSC treatment, and BM-MSC treatment. IPF was induced by administering bleomycin, followed by UC-MSC or BM-MSC treatment on day 14. Lung tissues and bronchoalveolar lavage fluid were collected on day 42 for analysis. The results demonstrated that UC-MSCs were more effective than BM-MSCs in reducing mortality, alleviating body weight loss, and reversing lung damage. UC-MSC treatment significantly decreased profibrotic markers like TGF-*β*, *α*-SMA, and hydroxyproline, as well as proinflammatory cytokines such as TNF-*α* and IL-6. In contrast, BM-MSCs provided limited protective effects and showed partial reductions in some indicators but did not significantly impact key markers. Collectively, these findings highlight the superior anti-fibrotic and anti-inflammatory efficacy of UC-MSCs compared with BM-MSCs, supporting the potential relevance of UC-MSCs as a promising cell source for future MSC-based therapeutic strategies.

## 1 Introduction

Pulmonary fibrosis encompasses a wide-ranging spectrum of lung disorders that are distinguished by the excessive deposition of fibrotic tissue in lung parenchyma [1], 2]. The buildup of fibrotic tissue causes permanent scarring and rigidity of the lungs and ultimately a gradual decline in respiratory function, including decreased lung compliance and impaired gas exchange [3], 4]. Idiopathic pulmonary fibrosis (IPF) is the most prevalent and severe form of chronic progressive fibrosing interstitial pneumonia, primarily affecting older adults [5], 6]. Although the pathogenesis of IPF remains unclear, several risk factors have been identified, including genetic disorders, environmental and occupational exposure, and the use of medications containing bleomycin, amiodarone, or methotrexate [6], [7], [8]. Leukocyte activation and infiltration play a critical role in collagen deposition and fibrosis formation within the pathogenesis of IPF, primarily through the expression of proinflammatory cytokines and chemokines [9], 10]. This process underscores the major influence of immune response mechanisms on the development and progression of IPF, highlighting the complex interaction between inflammatory mediators and fibrotic pathways [11], 12].

Mesenchymal stem cells (MSCs) are multipotent stromal cells that have gained considerable attention for their ability to differentiate into various cell types, including osteocytes, chondrocytes, and adipocytes, which has potential implications in regenerative medicine [13], 14]. Two prominent sources of MSCs include those that are bone marrow-derived (BM-MSCs) and umbilical cord-derived (UC-MSCs) [15], [16], [17]. The capacity of BM-MSCs and UC-MSCs to reduce IPF mortality rates has been extensively studied [18], 19]. BM-MSCs and UC-MSCs can improve pulmonary histopathology and respiratory function in IPF models through immunomodulatory effects, which include downregulation of leukocyte infiltration and the generation of proinflammatory cytokines. BM-MSCs and UC-MSCs have both exhibited promise for treating IPF via their antifibrotic properties [19], 20]. However, few comparative studies on their efficacy and mechanisms have been conducted. Although both types of MSCs may be effective, their distinct effects have not been thoroughly explored. Investigating the therapeutic potential of BM-MSCs and UC-MSCs in IPF models is crucial for identifying optimal treatment strategies and improving outcomes for patients with pulmonary fibrosis.

## 2 Materials and methods

### 2.1 Animal model and housing conditions

In this research, male BALB/c mice, aged 7–8 weeks, were acquired from the National Center for Biomodels (Taipei, Taiwan). The mice were housed in the Animal Science Center at Chung Shan Medical University in Taichung, Taiwan, in a specific pathogen-free environment. The facility maintained a controlled ambient temperature ranging from 23 to 25 °C and consistent humidity levels to ensure optimal living conditions. The mice were housed in standard laboratory cages and provided with a nutritionally balanced laboratory diet, along with unlimited access to fresh water. All experimental procedures involving animals, particularly those related to the induction of bleomycin-induced pulmonary fibrosis, were conducted following ethical guidelines.

**Ethical Approval:** The research related to animal use has been complied with all the relevant national regulations and institutional policies for the care and use of animals, and has been approved by the Institutional Animal Ethics Committee of Chung Shan Medical University (Approval No. 2429).

### 2.2 Animal experimental design

#### 2.2.1 Preparation and characterization of MSCs

In this study, we primarily aimed to investigate the therapeutic efficacy of UC-MSCs and BM-MSCs in mice with bleomycin-induced pulmonary fibrosis. The UC-MSCs and BM-MSCs were generously provided by BIONET Lab (Taipei, Taiwan). Human UC-MSCs were isolated from full-term umbilical cords donated by healthy women at 30 years old following informed consent. Cords from the female body were processed within 24 h post-delivery. After disinfection with 75 % ethanol and washing with DPBS (Gibco, Invitrogen, NY, USA), blood vessels were removed and Wharton’s jelly was minced (0.5–1 mm^3^). Tissue fragments were cultured in *α*-MEM (Gibco, Invitrogen, NY, USA) with 5 % UltraGRO™ (AventaCell, BioMedical, Atlanta, GA, USA) and antibiotics, at 37 °C and 5 % CO_2_. Medium was changed every 3–4 days. Upon confluence, cells were passaged using TrypLE™ Express (Gibco, Invitrogen, NY, USA) and expanded. Passage 3 (P3) cells were cryopreserved in CryoStor CS10 (BioLife Solutions, Bothell, WA, USA) and stored in vapor-phase liquid nitrogen at −190 °C. Human BM-MSCs were obtained from healthy donors (24–42 years) via iliac crest aspiration. Bone marrow was obtained from the iliac crest, which represents the standard and clinically established anatomical source for BM-MSC isolation, even in healthy donors. Mononuclear cells were isolated by Ficoll-Paque density centrifugation and cultured in *α*-MEM with 10 % FBS (Gibco, Invitrogen, NY, USA) and antibiotics. Non-adherent cells were removed after 48–72 h. Adherent MSCs were expanded and used at P3 for experiments. Inclusion criteria for both UC-MSC and BM-MSC donors included absence of chronic inflammatory, autoimmune, infectious, or malignant diseases. Exclusion criteria included a history of systemic illness, long-term medication use, or abnormal laboratory findings. All procedures were conducted under GMP-compliant conditions at BIONET Lab.

#### 2.2.2 Animal experimental design and treatment

**Figure 1:**
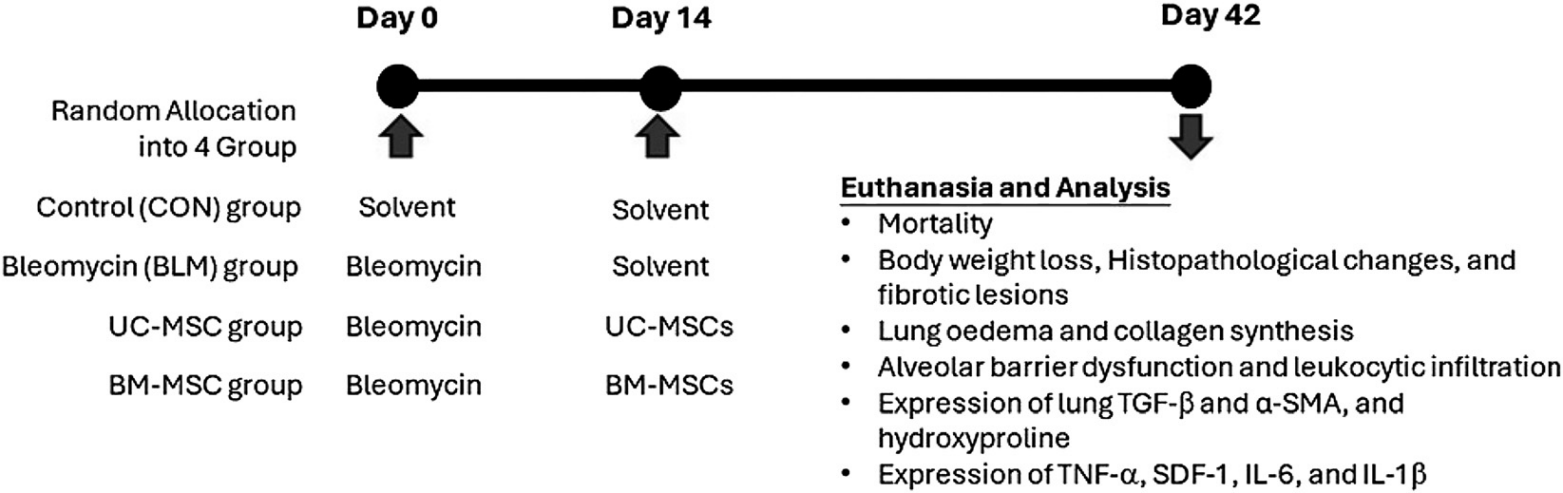
Flowchart of experimental procedures applied to bleomycin-induced IPF animal model treated with UC-MSCs or BM-MSCs.

A total of 24 male BALB/c mice were randomly allocated into 4 groups: a control (CON) group, administered intratracheal saline on days 0 and 14; a bleomycin (BLE) group, administered 5 mg/kg intratracheal bleomycin (Sigma-Aldrich, Grand Island, NY, USA) on day 0 and saline on day 14; a UC-MSC group, administered bleomycin on day 0 and 1 × 10^6^ UC-MSCs through intratracheal administration on day 14; and a BM-MSC group, administered bleomycin on day 0 and 1 × 10^6^ BM-MSCs through intratracheal administration on day 14. To ensure the welfare of animals, predefined humane endpoints were established to reduce suffering and comply with ethical standards. Animals were subject to daily monitoring, and any mouse that exhibited severe weight loss (greater than 15 % of baseline body weight), a significant reduction in food intake (less than 40 % of normal intake sustained over a period of seven consecutive days), or persistent anorexia (lasting longer than 48 h) was considered for early euthanasia. Additional criteria for evaluation included the presence of coarse fur texture, accompanied by clinical signs of distress, such as hunching, lethargy, or abnormal behavior. Should these symptoms persist despite supportive interventions, humane euthanasia was conducted prior to the predetermined endpoint of the study. On day 42, the experiment concluded, and all mice were euthanized with Zoletil^®^ (Vibac Laboratories, Carros, France) at 24 mg/kg and xylazine at 6 mg/kg through intramuscularly administration for sample collection (Figure 1). Mice were sacrificed on day 42 after bleomycin administration. This timepoint was selected to evaluate the chronic fibrotic phase of bleomycin-induced pulmonary fibrosis, at which acute inflammatory responses have largely subsided and stable fibrotic remodeling is established. Lung tissues were collected for further histological, biochemical, and molecular analyses to measure fibrosis, proinflammatory responses, and leukocyte infiltration. Bronchoalveolar lavage fluid (BALF) was collected immediately after euthanasia to evaluate leukocyte infiltration and analyze proinflammatory cytokine expression. The trachea was accessed and cannulated with a sterile intratracheal catheter, followed by gentle lung lavage using ice-cold phosphate-buffered saline for three washes. The recovered lavage fluid was centrifuged at 800*g* for 10 min at 4 °C to separate the supernatant and cell pellet. The supernatant was then stored at −80 °C for later analysis of pro-inflammatory cytokines, including TNF-*α*, IL-1β, and IL-6. Meanwhile, the cell pellet was used for leukocyte subtyping analysis through flow cytometry [21], 22].

### 2.3 Animal mortality monitoring

Following the administration of bleomycin to induce pulmonary fibrosis, animal mortality was monitored for a period of 42 days. A comprehensive assessment of physiological parameters, including heartbeat, respiration, ocular reflexes, and overall body movement, was conducted for determination of death.

### 2.4 Lung tissue weight and pulmonary index analysis

Upon euthanasia, mice lung tissues were excised and weighed to obtain their wet weights. The tissues were then dried in an oven maintained at 80 °C for 48 h to determine their dry weights. The degree of lung edema was quantitatively assessed according to the wet-to-dry weight ratio, calculated by dividing the wet weight by the dry weight. The pulmonary index, an indicator of lung hypertrophy relative to body weight, was calculated by dividing wet lung weight (mg) by body weight (g) [23], 24].

### 2.5 Lung tissue preparation

On the 42nd day after initial treatment administration, pulmonary tissues from each subject were excised and immediately submerged in 10 % neutral buffered formalin (Sigma-Aldrich, Saint Louis, Missouri, USA) to preserve integrity. After fixation, the tissues were embedded in paraffin, facilitating microtomy. Microsections of the paraffin-encased lungs were subsequently stained, and Hematoxylin-Eosin (H&E; Sigma-Aldrich, Saint Louis, Missouri, USA) stain or Masson’s trichrome stain techniques (Sigma-Aldrich, Saint Louis, Missouri, USA) were used to delineate cellular constituents and fibrous tissue. An automated microscopy system was used to assess the histological preparations for fibrotic alterations. Ten fields per slide were systematically examined to ensure exhaustive assessment of the tissue architecture. Fibrosis quantification was performed through Ashcroft scoring, ranging from 0 (unaltered lung architecture) to 8 (extensive fibrosis) [25]. The fibrous tissue areas within each field were also quantified. The proportion of fibrotic area was calculated relative to the total field area as (fibrotic area/total area) × 100 % to facilitate comparison between samples. This approach enabled quantitative appraisal of fibrotic evolution for the juxtaposition of fibrosis severity between the experimental cohorts [26].

### 2.6 Immunohistochemistry analysis

The lung sections underwent initial dewaxing and rehydration to facilitate their preparation for subsequent staining protocols. Subsequently, an EDTA-based antigen retrieval buffer was used to unmask antigens, improving antibody binding affinity. Hydrogen peroxide was used to mitigate non-specific staining caused by endogenous peroxidase activity. After blocking, the sections were incubated with primary antibodies that target tumor necrosis factor alpha (TNF*α*; Abcam, Cambridge, UK, Cat. No. ab183218; 1:200) and alpha smooth muscle actin (*α*-SMA; Abcam, Cambridge, UK, Cat. No. ab124964; 1:200), which serve as biomarkers for smooth muscle cells. Next, the slides were incubated with horseradish peroxidase-conjugated secondary antibodies. This step facilitates the binding of secondary antibodies (Jackson ImmunoResearch Laboratories, West Grove, PA, USA) to primary antibodies and thereby the detection of antigen-antibody complexes. Diaminobenzidine was used as a chromogen for visualization, yielding a brown precipitate at the locus of antigen expression. The processed slides were then meticulously analyzed with the Lion Heart FX automated microscope (BioTek Instruments, Winooski, VT, USA) [27].

### 2.7 Enzyme-linked immunosorbent assay

An enzyme-linked immunosorbent assay (ELISA) was performed to quantify the concentrations of collagen (Cayman Chemicals, Ann Arbor, MI, USA), hydroxyproline (Cayman Chemicals, Ann Arbor, MI, USA), stromal cell-derived factor 1 (SDF-1; Cayman Chemicals, Ann Arbor, MI, USA), and transforming growth factor beta (TGF-*β*; Cayman Chemicals, Ann Arbor, MI, USA) in the lung tissue samples. The samples were frozen, homogenized, and then centrifuged at a force of 5,000×*g* for 10 min at 4 °C, allowing the supernatants to separate. The supernatants were then used in ELISA kits designed for the measurement of hydroxyproline, SDF-1, and TGF-*β*. Concurrently, BALF samples were analyzed with the ELISA kits to determine the concentrations of TNF-*α*, IL-1β, and IL-6. All ELISA assays followed the manufacturer’s protocols to ensure the precision and repeatability of our results [28], 29].

### 2.8 Leukocyte infiltration in BALF

Leukocyte infiltration in BALF was assessed using flow cytometry. Following centrifugation, the cell pellet was resuspended in phosphate-buffered saline with 1 % bovine serum albumin. For immunophenotyping, cells were incubated with fluorochrome-conjugated antibodies targeting specific surface markers, including CD45 (pan-leukocyte marker; Biolegend, San Diego, CA, USA, Cat. No. 147712; 1:100), CD11b (myeloid marker; Biolegend, Cat. No. 101206; 1:100), Ly6G (neutrophil marker; Biolegend, Cat. No. 127606; 1:100), and CD3 (T lymphocyte marker; Biolegend, Cat. No. 100204; 1:100). Leukocytes were identified as CD45^+^ cells, granulocytes as CD45^+^CD11b^+^, neutrophils as CD45^+^Ly6G^+^, and lymphocytes as CD45^+^CD3^+^. Flow cytometric analysis included unstained controls and isotype-matched controls to define background fluorescence and establish gating strategies. Stained cells were resuspended in phosphate-buffered saline and analyzed with an Accuri C6 flow cytometer and software (BD Biosciences; Becton Dickinson, San Jose, CA, USA).

### 2.9 Statistical analysis

All statistical analyses were performed in SPSS (IBM, Armonk, NY, USA). Data are expressed as means ± standard deviations. The Shapiro-Wilk test was used to assess the normality of data distribution. For data with a normal distribution, a one-way analysis of variance (ANOVA) followed by Bonferroni’s post hoc test was applied for multiple group comparisons. The Kruskal-Wallis test was conducted for data that did not meet the normality assumption. When the Kruskal-Wallis test showed statistical significance, post hoc pairwise comparisons were conducted using Dunn’s test. Statistical significance was indicated as follows: comparisons between treatment groups and the CON group were denoted as ^#^ *P* < 0.05, comparisons with the BLE group were denoted as *P < 0.05, and comparisons with the UC-MSC group were denoted as ^$^ *P* < 0.05.

## 3 Results

### 3.1 Alleviation of mortality in bleomycin-induced IPF by UC-MSCs and BM-MSCs

**Figure 2:**
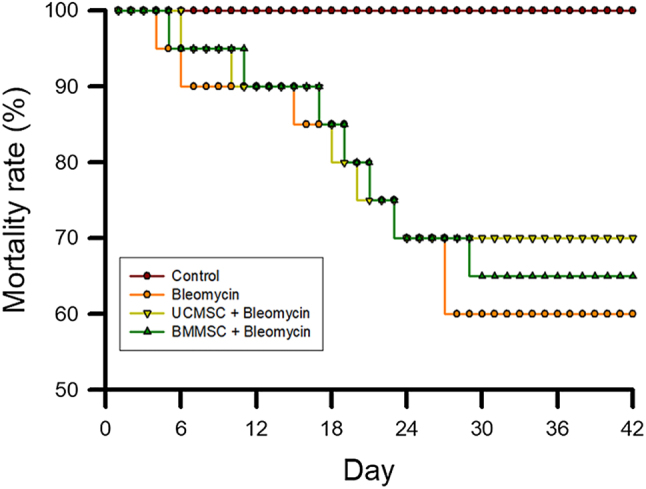
Alleviation of mortality in bleomycin-induced IPF by UC-MSCs and BM-MSCs.

To assess the efficacy of UC-MSCs and BM-MSCs in mitigating mortality in bleomycin-induced IPF, mice exposed to bleomycin were subsequently treated with UC-MSCs and BM-MSCs 14 days after exposure. Mortality rates were recorded daily for 42 days following bleomycin administration, allowing comprehensive evaluation of the therapeutic potential of MSC treatments for IPF. Mice in the BLE group exhibited severe mortality compared with that of the CON group. Additionally, UC-MSCs significantly surpassed BM-MSCs in reducing mortality rates (Figure 2).

### 3.2 Effect of UC-MSCs and BM-MSCs on body weight loss, histopathological changes, and fibrotic lesions

**Figure 3:**
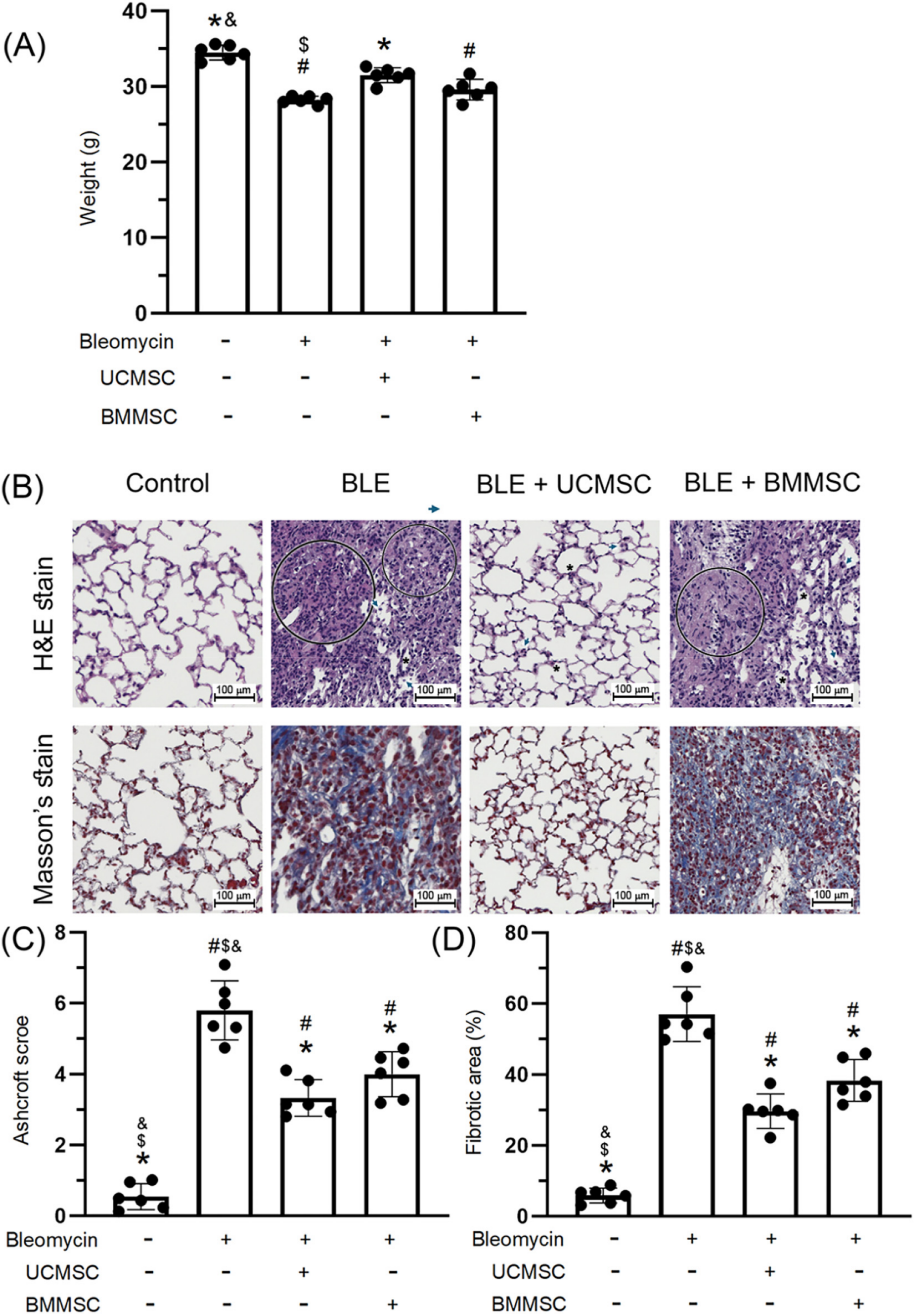
The impact of UC-MSCs and BM-MSCs on body weight loss, histopathological alterations, and fibrotic lesions. Panel (A) presented the variations in body weight. Panel (B) displayed the histopathological morphology of lung tissues, which was evaluated through H&E and Masson’s trichrome staining techniques. Scale bar = 100 μm. Circles indicate regions of pulmonary architectural disruption; asterisks denote alveolar wall thickening; arrowheads indicate leukocyte infiltration. Panel (C) depicted the Ashcroft score, while panel (D) highlighted the fibrosis-related areas. The data were expressed as mean ± standard deviation for six mice per experimental group. Statistical significance was defined for comparisons: ^#^ *P* < 0.05 versus CON group; *P < 0.05 versus BLE group; and ^$^ *P* < 0.05 versus UC-MSC group.

Our findings indicate that bleomycin triggered marked body weight loss (*P* < 0.05, Figure 3A). Treatment with UC-MSCs, but not with BM-MSCs, significantly decreased the weight loss induced by bleomycin (*P* < 0.05). These histopathological changes were assessed using H&E stain, as illustrated in Figure 3B. Histopathological changes, including destruction of the pulmonary structure, alveolar wall thickening, and leukocyte infiltration, were more pronounced in the BLE than in the CON group. Treatment with either UC-MSCs or BM-MSCs significantly improved histopathological changes and the Ashcroft score (*P* < 0.05, Figure 3C). Fibrotic lesion areas were assessed through Masson’s trichrome staining, as displayed in Figure 3B. Fibrotic lesions were more prevalent in the BLE group than the CON group. Notably, treatment with either UC-MSCs or BM-MSCs significantly improved fibrotic lesions (*P* < 0.05, Figure 3D). Both types of MSC exhibited antifibrotic properties and corresponding histopathological improvements. However, UC-MSCs demonstrated superior efficacy in mitigating fibrosis and restoring pulmonary structural integrity, as supported by quantitative scoring and histological analysis.

### 3.3 Effect of UC-MSCs and BM-MSCs on lung edema and collagen synthesis

**Figure 4:**
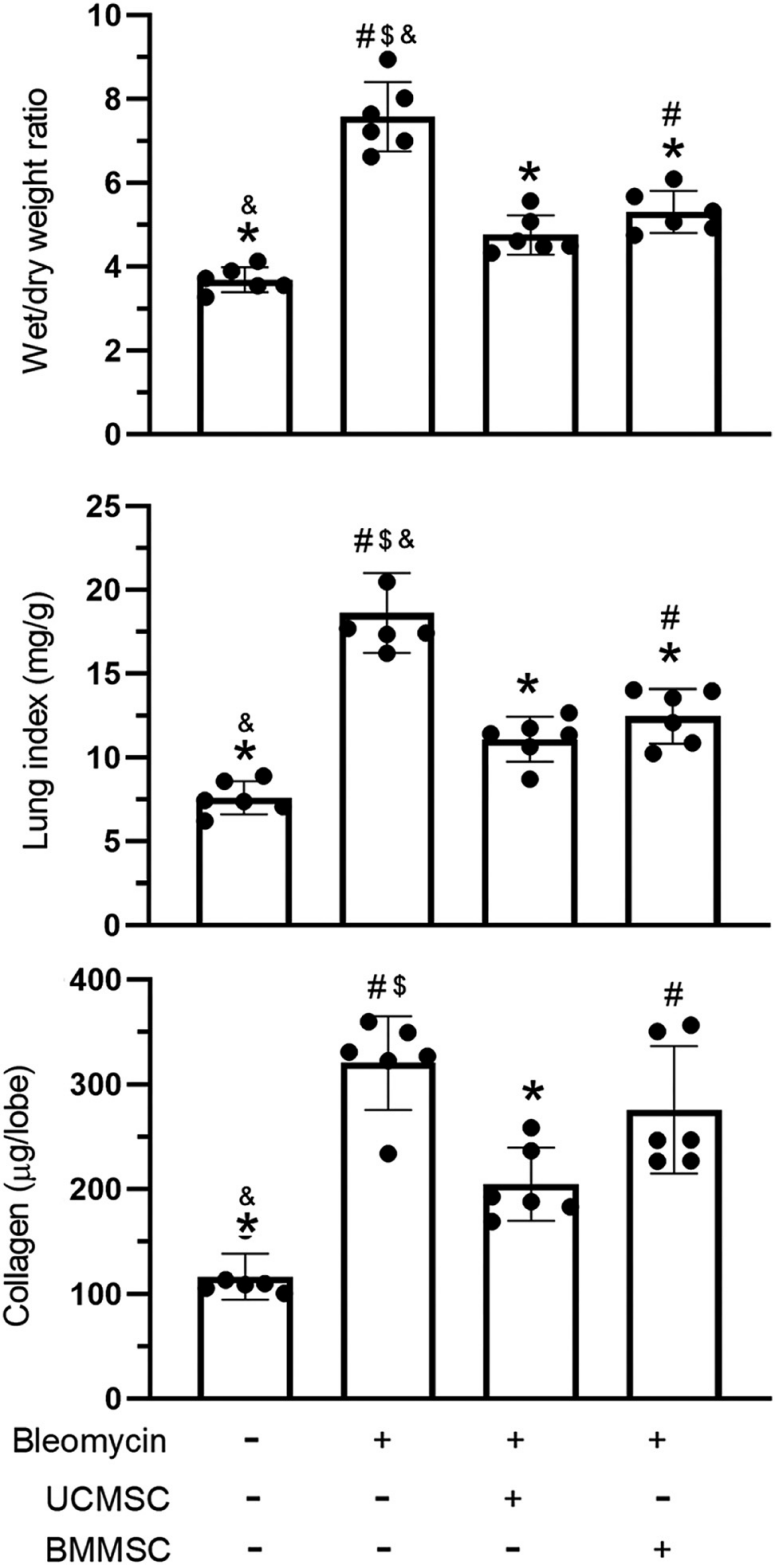
Effect of UC-MSCs and BM-MSCs on lung edema and collagen synthesis. The evaluated parameters included lung index, wet-to-dry weight ratio, and collagen synthesis. The results were presented as mean ± standard deviation, based on six mice per experimental group. Statistical significance was defined for comparisons: ^#^ *P* < 0.05 versus CON group; *P < 0.05 versus BLE group; and ^$^ *P* < 0.05 versus UC-MSC group.

The lung index and wet-to-dry weight ratio are key markers for assessment of lung edema in IPF models. Lung edema was markedly triggered by bleomycin administration and significantly improved after treatment with UC-MSCs or BM-MSCs (*P* < 0.05, Figure 4). Collagen expression was significantly elevated following the administration of bleomycin and markedly improved after treatment with UC-MSCs, whereas treatment with BM-MSCs did not yield similar results (*P* < 0.05, Figure 4).

### 3.4 Alleviation of alveolar barrier dysfunction and leukocytic infiltration following UC-MSC and BM-MSC intervention

**Figure 5:**
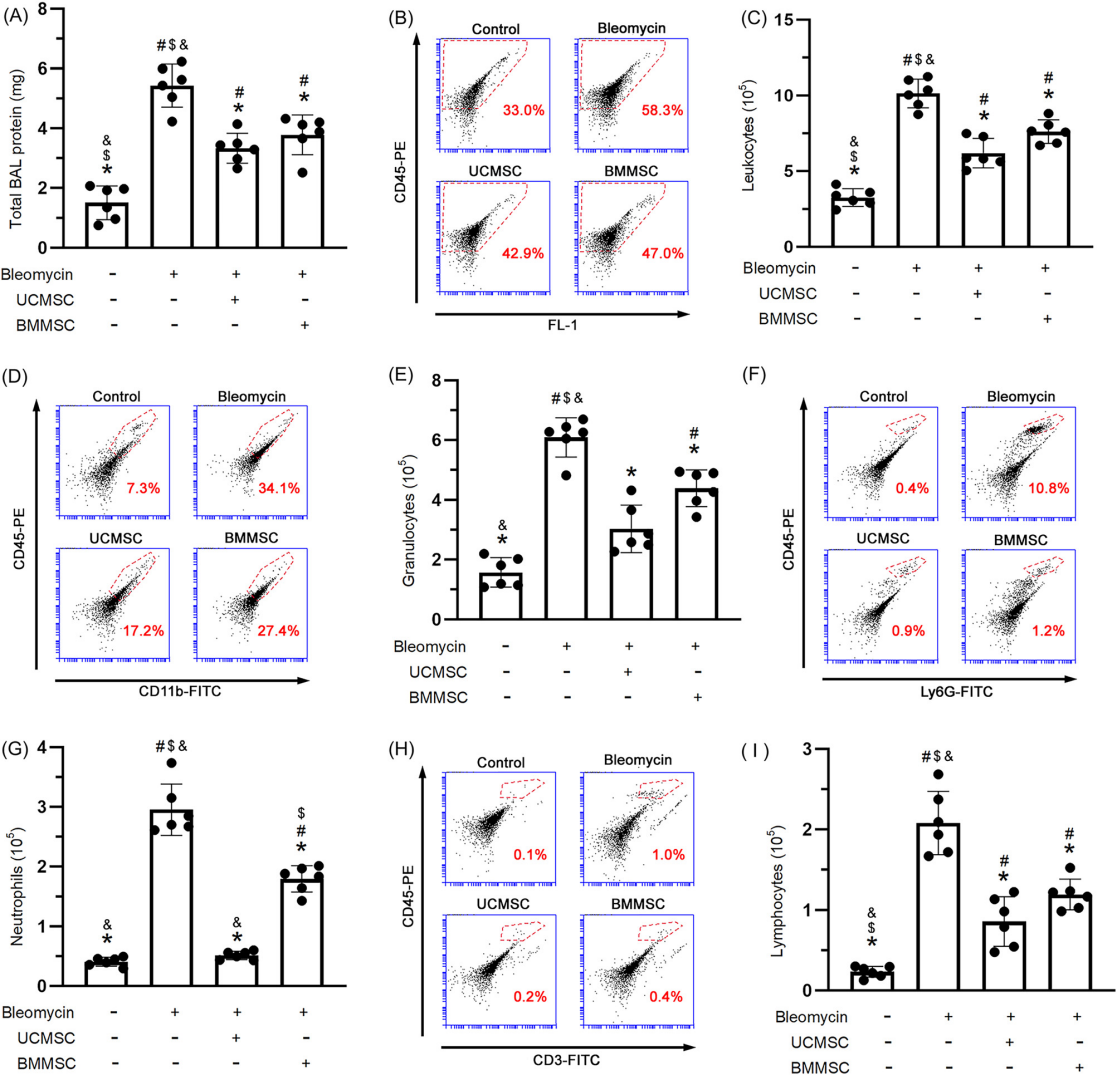
Alleviation of alveolar barrier dysfunction and leukocytic infiltration following UC-MSC and BM-MSC intervention. The evaluated parameters included leakage of protein (A) and infiltration of leukocytes (C), including granulocytes (E), neutrophils (G), and lymphocytes (I). The flow cytometry dot plots illustrated the cellular distribution of leukocytes (B), granulocytes (D), neutrophils (F), and lymphocytes (H) in the BALF. The results were presented as mean ± standard deviation, based on six mice per experimental group. Statistical significance was defined for comparisons: ^#^ *P* < 0.05 versus CON group; *P < 0.05 versus BLE group; and ^$^ *P* < 0.05 versus UC-MSC group.

Alveolar barrier dysfunction was assessed on the basis of protein concentrations in BALF. Bleomycin administration resulted in significant alveolar barrier dysfunction (*P* < 0.05, Figure 5A) that was significantly improved by treatment with UC-MSCs (*P* < 0.05) but not treatment with BM-MSCs (Figure 5A). Alveolar barrier dysfunction allows various leukocyte populations, including granulocytes, neutrophils, and lymphocytes, to infiltrate the lung tissue, contributing to inflammatory response. The infiltration of leukocytes (Figure 5B and C), including granulocytes (Figure 5D and E), neutrophils (Figure 5F and G), and lymphocytes (Figure 5H and I), was significantly elevated following bleomycin administration (*P* < 0.05). Notably, treatment with either UC-MSCs or BM-MSCs significantly improved leukocyte infiltration into fibrotic lesions (*P* < 0.05, Figure 5).

### 3.5 Reduced expression of lung TGF-*β*, a-SMA, and hydroxyproline via UC-MSCs

**Figure 6:**
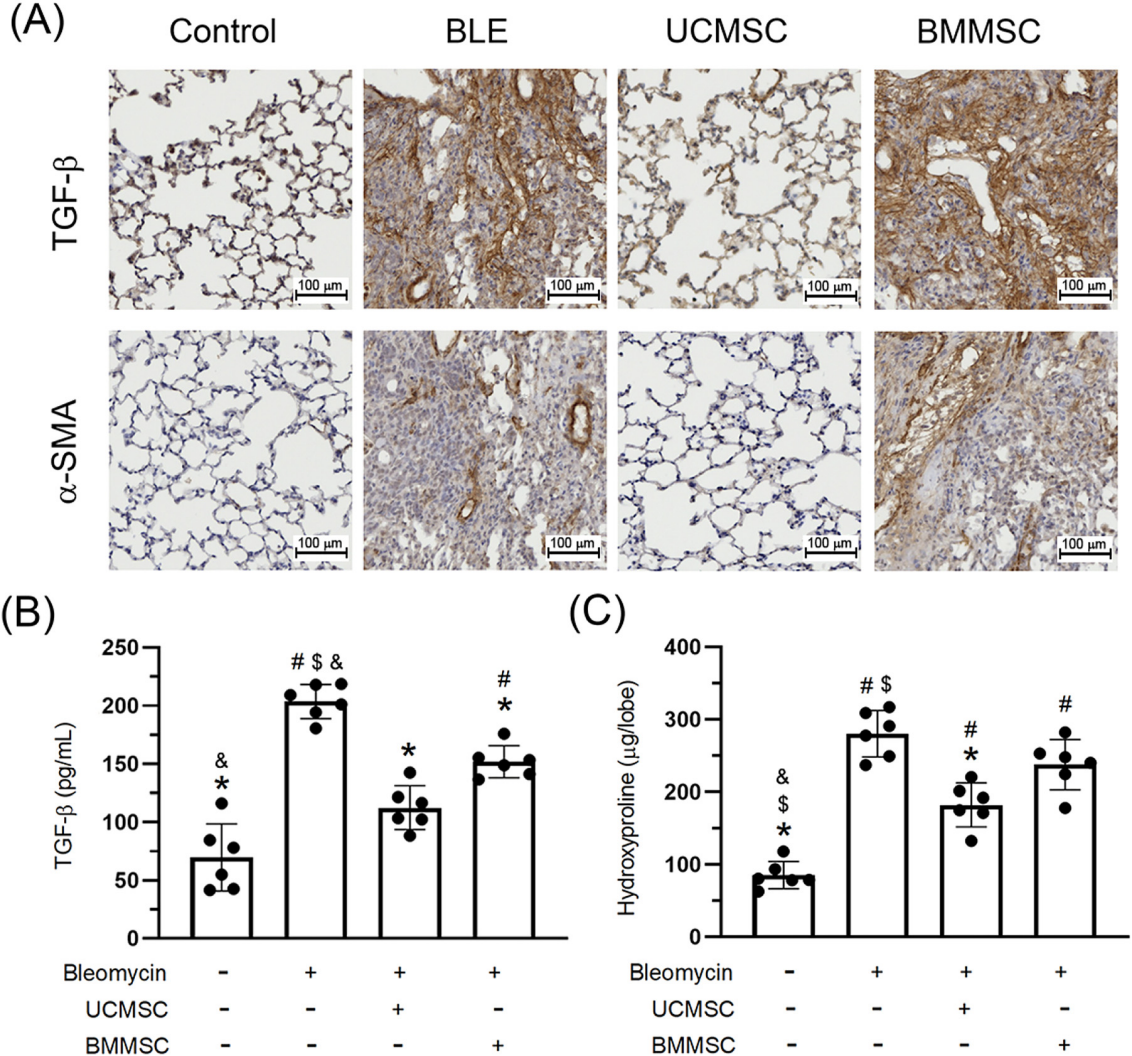
Effect of UC-MSCs and BM-MSCs on the expression of lung TGF-β, a-SMA, and hydroxyproline. Panel (A) presented the expression of lung TGF-β and a-SMA, evaluated through Immunohistochemistry analysis. Scale bar = 100 μm. Panel (B) displayed the level of TGF-β expression. Panel (C) displayed the level of hydroxyproline expression. Statistical significance was defined for comparisons: ^#^ *P* < 0.05 versus CON group; *P < 0.05 versus BLE group; and ^$^ *P* < 0.05 versus UC-MSC group.

In our investigation of the pathological progression of pulmonary fibrosis, we observed elevated expression of the profibrotic markers TGF-*β* and *α*-SMA in the BLE group, consistent with the progressive fibrotic remodeling in the lung. Immunohistochemical analysis indicated that UC-MSCs treatment significantly reduced tissue-level TGF-*β* and *α*-SMA staining, while BM-MSCs showed only limited reductions (Figure 6A). However, biochemical quantification via ELISA (Figure 6 B and C) demonstrated that both MSC treatments significantly reduced TGF-*β* levels in lung homogenates (*P* < 0.05), while only UC-MSCs significantly reduced hydroxyproline content (*P* < 0.05), a surrogate marker of collagen accumulation. Overall, these results reinforce the superior antifibrotic efficacy of UC-MSCs, which not only suppressed TGF-*β* signaling but also significantly reduced collagen accumulation and myofibroblast activity. In contrast, BM-MSCs provided limited improvements.

### 3.6 Effect of UC-MSCs and BM-MSCs on TNF-a, SDF-1, IL-6, and IL-1β expression

**Figure 7:**
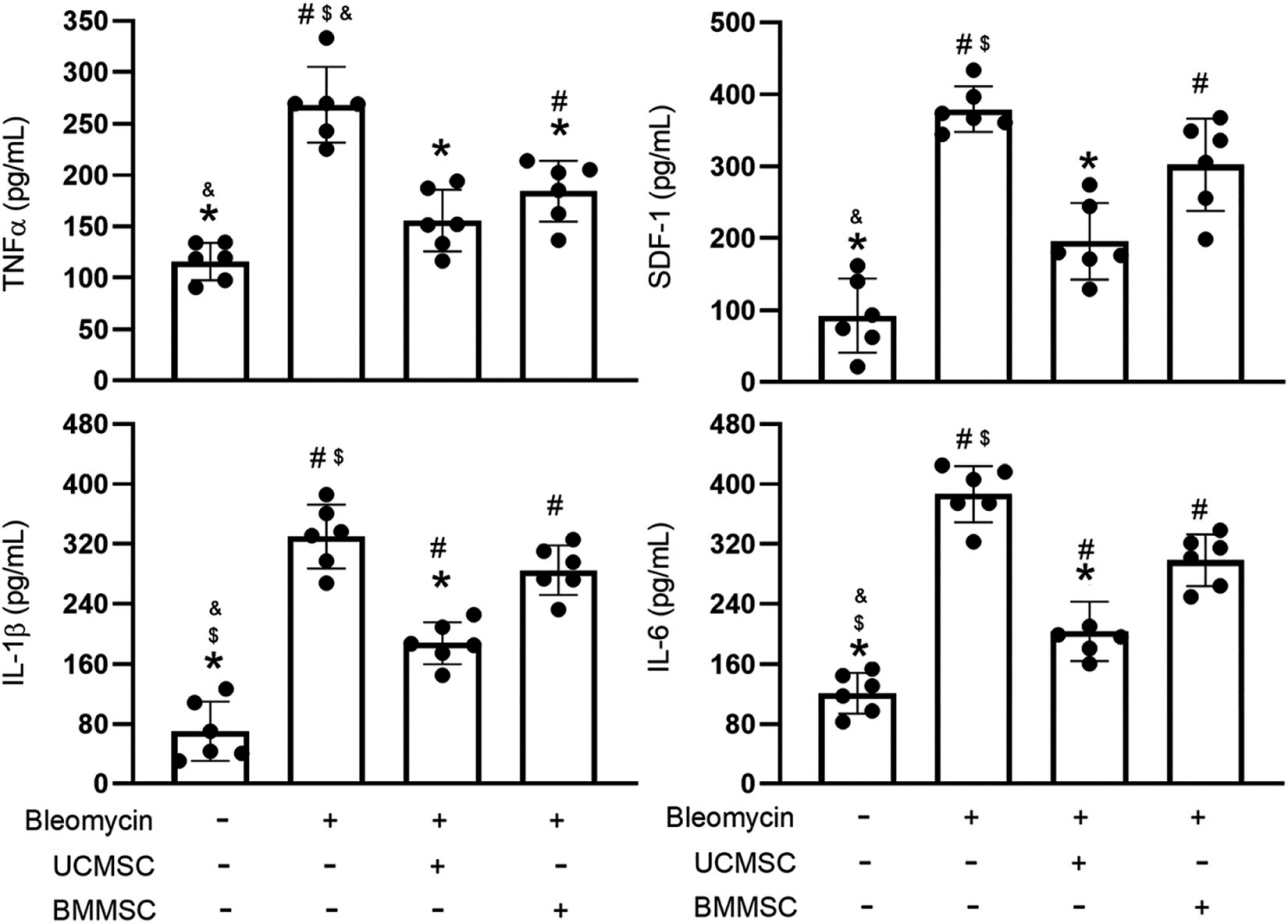
Effect of UC-MSCs and BM-MSCs on TNF-α, SDF-1, IL-6, and IL-1β expression. The evaluated parameters included TNF-α, SDF-1, IL-6, and IL-1β expression. The results were presented as mean ± standard deviation, based on six mice per experimental group. Statistical significance was defined for comparisons: ^#^ *P* < 0.05 versus CON group; *P < 0.05 versus BLE group; and ^$^ *P* < 0.05 versus UC-MSC group.

ELISA analysis was performed to determine TNF-*α*, SDF-1, IL-6, and IL-1β levels in BALF (Figure 7). TNF-*α*, SDF-1, IL-6, and IL-1β expression were greater in the BLE group than in the CON group (*P* < 0.05, Figure 7). Notably, treatment with either UC-MSCs or BM-MSCs significantly improved TNF-*α* expression (*P* < 0.05, Figure 7). Additionally, treatment with UC-MSCs but not BM-MSCs significantly improved SDF-1, IL-6, and IL-1β expression (*P* < 0.05, Figure 7).

## 4 Discussion

IPF is a severe lung disease characterized by the gradual thickening and stiffening of lung tissue due to scarring, known as fibrosis. This scarring impairs the lungs’ ability to function properly, causing progressive breathing difficulties and other symptoms, such as shortness of breath, a persistent dry cough, and fatigue. The exact cause of IPF remains unknown. Studies have demonstrated that IPF predominantly affects older adults between the ages of 70 and 75 and is relatively uncommon in individuals younger than 50. Those diagnosed with IPF generally have a survival expectancy of 2–5 years after diagnosis [30]. In Europe, the incidence of IPF ranges from 3 to 9/100,000 person-years, with higher rates in the U.K. and Sweden [31]. In the U.S., the incidence is estimated to be 6.8 to 16.3/100,000 person-years, whereas Japan reports lower rates of approximately 1.2–4.6/100,000 person-years [32]. In the U.K., IPF incidence nearly doubled from 3.31/100,000 person-years in 1998 to 6.81 in 2008. Similarly, in the U.S., Medicare data indicated an increase in IPF incidence among adults aged 65 and older from 27.9/100,000 person-years in 2001 to 63 in 2011 [33], 34]. The unknown etiology and heterogenous disease progression of IPF and its limited treatment options pose substantial challenges to effective management [35], 36]. To date, both UC-MSCs and BM-MSCs have exhibited promise as novel therapeutic approaches for IPF in animal and clinical trials, offering potential benefits through their immunomodulatory and regenerative properties [37], [38], [39], [40]. However, few comparative studies have been conducted on the mechanisms through which UC-MSCs and BM-MSCs treat IPF in animal models or the effectiveness of such treatments. In our IPF animal model, we observed that the mortality induced by bleomycin through intratracheal injection was comparable across all groups. However, the mortality rate was lower in groups receiving UC-MSCs and BM-MSCs from day 14–42 compared with the BLE group. In a previous study, intravenous injection of UC-MSCs from days 7 and 21 to 35 increased survival following bleomycin administration [41]. However, no evidence suggested that BM-MSCs improved the survival rate after bleomycin treatment. Additionally, our findings indicate that UC-MSCs were more effective than BM-MSCs in reducing mortality in mice with IPF induced by bleomycin.

The features of bleomycin-induced IPF in animal models include body weight loss, lung edema, fibrotic lesion formation, collagen synthesis, and histology changes [42], [43], [44]. Weight loss is often correlated with IPF severity and arises from multiple factors, including the reduced systemic inflammation and increased metabolic demands associated with tissue repair [45], 46]. The selection of day 42 as the experimental endpoint allowed assessment of sustained fibrotic remodeling rather than transient inflammatory changes. At this stage, collagen deposition, myofibroblast accumulation, and structural distortion of lung architecture are well established, providing a stringent framework to evaluate long-term antifibrotic efficacy of MSC-based interventions. In our study, beyond 14 days after bleomycin administration, the histopathological changes observed in H&E-stained sections were dominated by advanced fibrosis and architectural distortion. Dense collagen deposition replaced normal lung parenchyma, obliterating functional alveoli and causing marked Masson’s staining of fibrotic regions. The alveolar walls collapsed, contributing to the loss of gas exchange surfaces. Although overall inflammation subsided, chronic immune cells, including macrophages and lymphocytes, persisted in the fibrotic regions, sustaining low-grade inflammation. Vascular remodeling was also apparent, with thickened vessel walls and perivascular fibrosis contributing to disease severity [47], 48]. Fluid accumulation disrupted alveolar-capillary integrity, contributing to hypoxia and amplifying the inflammatory cascade. Although edema partially resolved during the fibrotic phase, residual low-grade edema sustained fibroblast activation and further collagen deposition, perpetuating the fibrotic cycle [47], 48]. UC-MSCs administered intravenously reversed body weight loss, the formation of fibrotic lesions, and histological changes caused by bleomycin on days 7 and 21, with subjects euthanized on day 35 [41]. When administered via intravenous and tracheal spray, UC-MSCs also reversed similar concerns on day 14, with subjects euthanized on day 28 [49]. UC-MSCs administered intratracheally corrected these problems on days 7 or 10, with subjects euthanized on day 14 [50], 51]. Intratracheal administration reversed the negative effects of bleomycin administration on day 21, with subjects euthanized on day 49 [52], 53].

BM-MSCs administered intravenously have exhibited promise in reversing the effects of bleomycin. BM-MSCs were found to reverse fibrotic lesion formation, collagen synthesis, and histological changes on day 28, with subjects euthanized on day 56 [54]. They also reversed fibrotic lesions and histological changes by day 14 [54]. In another study, BM-MSCs mitigated fibrotic lesions after 7 days, with subjects euthanized on day14, and reversed lesion outbreak by day 21, with subjects euthanized on day 49 [55], 56]. Additionally, BM-MSCs caused reversal of lesions and histological changes by day 28, with subjects euthanized on day 56 [57]. Within 6–8 h, BM-MSCs also improved overall condition, after which subjects were euthanized on day 21 [58]. Finally, fibrotic lesions and histological changes were reversed by days 1, 3, and 6, with sacrifice on day 28 [59]. In our study, bleomycin-induced pulmonary injury led to significant body weight loss, lung edema, the development of fibrotic lesions, and structural damage to lung tissue. Both UC-MSCs and BM-MSCs mitigated the pathological changes caused by bleomycin; however, we observed notable differences in their therapeutic effectiveness. Specifically, mice treated with UC-MSCs experienced a significant recovery in body weight by day 42, whereas those in the BM-MSC group did not demonstrate comparable improvements. This indicates a more pronounced systemic protective effect associated with UC-MSCs. Both types of MSC treatments demonstrated a significant reduction in alveolar wall thickening, fibrotic deposition, and inflammatory infiltration, as indicated by improved Ashcroft scores and decreased percentages of fibrotic area. However, representative images from H&E and Masson’s trichrome staining showed that BM-MSCs were less effective in restoring normal alveolar architecture compared to UC-MSCs. This discrepancy likely reflects the differing abilities of these two MSC types in modulating tissue remodeling. While BM-MSCs exhibited partial antifibrotic effects sufficient to enhance quantitative fibrosis measures, they were not as effective in reversing the disorganized structural integrity associated with bleomycin-induced injury. Our findings suggest that both UC-MSCs and BM-MSCs provide significant antifibrotic benefits; however, UC-MSCs demonstrate superior therapeutic effects, especially in restoring pulmonary structure and facilitating systemic recovery. This supports the notion that UC-MSCs may possess enhanced reparative potency in the treatment of bleomycin-induced pulmonary fibrosis.

Dysfunction of the alveolar barrier represents a major pathological hallmark of IPF progression induced by bleomycin. This dysfunction is characterized by increased epithelial permeability, which reflects the compromised integrity of tight junctions. Collectively, these changes enhance the susceptibility of lung tissue to injury, which is a critical contributor to acute inflammation and the development of chronic fibrotic remodeling [60], [61], [62]. Damage to the delicate alveolar barrier facilitates the infiltration of immune cells like lymphocytes and granulocytes, which comprise macrophages and neutrophils, into the alveolar space. The influx of immune cells triggers a substantially heightened inflammatory response characterized by increased signaling molecules and other inflammatory agents. These findings are consistent with those of prior studies, indicating that a compromised alveolar barrier not only initiates acute inflammation but also plays a critical role in the subsequent development of chronic fibrotic remodeling, causing long-term changes in lung tissue structure and function [9], 11]. UC-MSCs administered intravenously or intratracheally have been demonstrated to reverse the bleomycin-induced infiltration of total cells, including macrophages, neutrophils, and lymphocytes, into BALF on day 10, with subjects euthanized on day 14 [50]. UC-MSCs also reversed myeloperoxidase activity, a granulocyte marker in lung tissue, on day 7 [51]. Additionally, on day 21, BM-MSCs administered intravenously promoted macrophages and inhibited production of lymphocytes in the alveolar space due to bleomycin, with subjects euthanized on day 49 [56]. Our findings indicated that the administration of bleomycin significantly disrupted alveolar barrier function, increasing leukocyte infiltration. Following a 28-day treatment with UC-MSCs and BM-MSCs after 14 days of bleomycin administration, alveolar barrier recovery and reduction in leukocyte infiltration were observed on day 42. Crucially, our results revealed that UC-MSCs were more effective than BM-MSCs in alleviating bleomycin-induced neutrophil infiltration in mice with IPF.

Bleomycin-induced IPF models have affirmed the pivotal role of TGF-*β*, *α*-SMA, and hydroxyproline as markers of disease activity [34], 63]. The interaction between TGF-*β*, *α*-SMA, and hydroxyproline highlights a self-reinforcing loop in IPF pathogenesis. Elevated TGF-*β* levels initiate the fibroblast to myofibroblast transition, as evidenced by increased *α*-SMA expression. The resultant overproduction of extracellular matrix, indicated by hydroxyproline accumulation, perpetuates tissue stiffening and further activation of TGF-*β* [64]. This cycle underscores the need for interventions that disrupt fibrogenic feedback. Emerging therapies that target TGF-*β* signaling, such as euthanized antibodies and small molecule inhibitors, have attenuated both *α*-SMA expression and hydroxyproline deposition in preclinical models, thereby mitigating fibrosis progression [65]. UC-MSCs administered intravenously reversed bleomycin-induced expression of TGF-*β* and *α*-SMA on days 7 and 21, with subjects euthanized on day 35 [41]. Additionally, UC-MSCs administered via tracheal spray reversed *α*-SMA expression on day 14, with subjects euthanized on day 28 [49]. UC-MSCs administered intratracheally reversed TGF-*β* and *α*-SMA expression on day 7, with subjects euthanized on day 14 [51], and *α*-SMA and hydroxyproline expression on day 21, with subjects euthanized on day 49 [52], 53]. For BM-MSCs, intravenous administration reversed TGF-*β* and *α*-SMA expression on day 28, with subjects euthanized on day 56 [48]. BM-MSCs also reversed hydroxyproline expression on day 7 [55], inhibited TGF-*β* expression and hydroxyproline on day 21 [56], and reversed multiple expressions within 6–8 h, with subjects euthanized on day 21 [58]. Reversals of TGF-*β* expression were also observed on days 1, 3, and 6, with subjects euthanized on day 28 [59].

The results of our study indicate that administration of bleomycin for 14 days and subsequently UC-MSCs or BM-MSCs, culminating in lung harvesting on day 42, significantly increased the expression levels of TGF-*β*, *α*-SMA, and hydroxyproline in the lung tissue. In murine models of bleomycin-induced IPF, the administration of UC-MSCs decreased the expression of TGF-*β*, *α*-SMA, and hydroxyproline in the lung tissue. Conversely, BM-MSCs reduced TGF-*β* and *α*-SMA expression but did not affect hydroxyproline levels. Our findings likewise indicated that the reduction in TGF-*β* and *α*-SMA expression was more pronounced with the administration of UC-MSCs than BM-MSCs. Our results demonstrate that bleomycin administration triggered a robust fibrotic response, as evidenced by elevated levels of TGF-*β*, *α*-SMA, and hydroxyproline in lung tissues by day 42. Treatment with UC-MSCs markedly attenuated the expression of all three fibrosis-associated markers, indicating effective suppression of profibrotic signaling, myofibroblast activation, and collagen deposition. In contrast, BM-MSCs led to a modest reduction in TGF-*β* and *α*-SMA expression but failed to significantly decrease hydroxyproline levels, suggesting their impact on extracellular matrix accumulation is limited. Notably, the antifibrotic effects of UC-MSCs were consistently superior to those of BM-MSCs across both immunohistochemical and biochemical analyses. The more pronounced suppression of TGF-*β* and *α*-SMA by UC-MSCs implies a stronger capacity to modulate fibrotic remodeling and inhibit myofibroblast differentiation. Conversely, the partial efficacy observed in the BM-MSC group may reflect suboptimal paracrine regulation, which, while sufficient to downregulate certain profibrotic mediators, did not translate into a significant reduction in collagen deposition, as indicated by unchanged hydroxyproline levels.

Proinflammatory cytokines, including TNF-*α*, SDF-1, IL-6, and IL-1β, play a pivotal role in the inflammatory and fibrotic progression of bleomycin-induced IPF. This progression includes recruitment and activation of leukocytes, including granulocytes and lymphocytes, due to the peripheral circulation induced by TNF-*α*, SDF-1, IL-6, and IL-1β [66], 67]. Proinflammatory cytokines also exacerbate apoptosis in alveolar epithelial cells, promote fibroblast activation, and disrupt the alveolar barrier, contributing to fibrotic tissue remodeling [68], 69]. Recent research has demonstrated that UC-MSCs, administered intravenously or intratracheally, can reverse the expression of chemokines induced by bleomycin by day 10, with subjects euthanized on day 14 [60]. BM-MSCs, when administered intravenously, were found to reverse bleomycin-induced TNF-*α* and IL-6 expression by day 28, with subjects euthanized on day 56 [54]. BM-MSCs also successfully reversed TNF-*α* and IL-6 levels by day 14 (with subjects euthanized on day 28) [54] and reduced IL-6 and IL-1β levels by day 7 (with subjects euthanized on day-14) [55]. Our study presents major findings regarding the effects of bleomycin treatment. After administration of bleomycin on day 14 and subsequent UC-MSCs or BM-MSCs, we harvested lung tissue from specimens on day 42. We observed a notable increase in proinflammatory cytokines, specifically TNF-*α*, SDF-1, IL-6, and IL-1β, in the BALF. In murine models of bleomycin-induced IPF, treatment with UC-MSCs notably improved the expression of these cytokines within lung tissue. By contrast, although BM-MSCs decreased TNF-*α* levels, they did not significantly affect SDF-1, IL-6, or IL-1β levels. Crucially, the reduction in proinflammatory cytokines associated with UC-MSCs was significantly more pronounced than that observed with BM-MSCs.

The findings of this study demonstrate the superior efficacy of UC-MSCs over BM-MSCs in attenuating the expression of proinflammatory cytokines, including TNF-*α*, SDF-1, IL-6, and IL-1β, in our bleomycin-induced IPF model. In contrast to BM-MSCs, UC-MSCs from umbilical cord-derived Wharton’s jelly displayed a markedly high proliferative capacity and low immunogenicity [70], 71], as evidenced by their inhibition of activated lymphocyte proliferation and modulation of cytokine secretion profiles. UC-MSCs are derived from perinatal tissues, providing them with greater youthfulness that bolsters their proliferation and genomic stability. By contrast, BM-MSCs are sourced from adult donors; thus, the effects of aging diminish their proliferation and therapeutic effectiveness [72], 73]. BM-MSCs exhibit a proinflammatory response when exposed to lipopolysaccharide or polyinosinic:polycytidylic acid, causing upregulation of proinflammatory genes, including IL1A, IL1B, IL7, IL8, ICAM1, IL12A, NLRP3, and TLR1 and TLR4 IL1A, IL1B, IL8, ICAM1, and NLRP3. By contrast, UC-MSCs preferentially upregulate immunosuppressive markers, such as adhesion molecules, CD274 (PD-L1), CD255 (TWEAK), TLR3, EGFR, and CD46, which suppress T cell activation and promote apoptosis-mediated immune modulation [74]. Additionally, UC-MSCs secrete more IL-10, facilitating macrophage polarization toward the M2 phenotype and reducing fibrosis [75], 76]. The superior efficacy of UC-MSCs over BM-MSCs in reducing TNF-*α*, SDF-1, IL-6, and IL-1β expression in pulmonary fibrosis likely stems from the stronger immunosuppressive phenotype, distinct cytokine secretion profiles, and greater interactions with immune cells of US-MSCs.

Several molecular pathways, including those associated with TGF-*β*1, NF-κB, and Wnt/β-catenin, may contribute to the superior therapeutic potential of UC-MSCs over BM-MSCs in modulating key signaling pathways involved in bleomycin-induced IPF. UC-MSCs mitigate fibrosis by inhibiting TGF-*β*1, a crucial driver of fibroblast activation and extracellular matrix deposition [38], 77]. Although the studies reviewed herein do not explicitly discuss the suppression of Smad2/3 phosphorylation by UC-MSCs, hepatocyte growth factor, bone morphogenetic protein-7, and other factors derived from UC-MSCs may reduce fibroblast-to-myofibroblast differentiation [38], 78]. UC-MSCs exhibit a superior capacity for TGF-*β* modulation to BM-MSCs, which is likely attributable to their distinctive immunomodulatory properties and adaptability to inflammatory microenvironments [78]. The NF-κB signaling pathway is a central regulator of inflammatory responses in IPF and plays a well-established role in the bleomycin-induced experimental model. Activation of NF-κB promotes the transcription of multiple pro-inflammatory and profibrotic mediators, thereby linking innate immune activation to fibroblast activation and extracellular matrix deposition. Among its downstream effectors, IL-1β is a key cytokine implicated in sustained pulmonary inflammation and fibrotic progression, contributing to inflammatory cell recruitment and profibrotic signaling within the lung microenvironment. Thus, modulation of NF-κB activity and IL-1β production provides an important mechanistic context for understanding the anti-inflammatory and antifibrotic effects observed in this study. The NF-κB pathway drives myofibroblast activation and proinflammatory cytokine expression, including TNF-*α*, IL-6, and IL-1β, which in turn drive chronic inflammation in IPF [79], [80], [81]. UC-MSCs exert anti-inflammatory effects by secreting PGE2 and TSG-6 to inhibit the NF-κB pathway [79], 80]. Studies have indicated that NF-κB inhibition can reduce macrophage-driven inflammation and modulate TGF-*β*1/MAPK signaling to alleviate fibrosis ^79,80,82^. BM-MSCs are likely derived from UC-MSCs, which demonstrate greater efficacy in modulating cytokine networks by increasing IFN-γ and IL-10 expression while suppressing TNF-*α* and IL-6 expression. Although BM-MSCs and UC-MSCs share certain anti-inflammatory properties, the unique composition of secretome in UC-MSCs and their effectiveness in targeting NF-κB-dependent pathways may explain their greater therapeutic efficacy in IPF [79], 80], 82]. Wnt/β-catenin signaling is integral to IPF pathogenesis given its role in promoting fibroblast activation, myofibroblast differentiation, and extracellular matrix deposition [83], 84]. UC-MSCs secrete Dickkopf-1 (DKK-1) and secreted frizzled-related protein-1, which bind to Wnt ligands or receptors to inhibit *β*-catenin stabilization [84], 85]. This inhibition disrupts the nuclear translocation of *β*-catenin and its transcriptional activation of profibrotic genes, including *α*-SMA, fibronectin, and collagen I [83], 86]. However, no existing evidence suggests that BM-MSCs improve IPF through the Wnt/β-catenin pathway. Although the benefits of UC-MSCs are clear, their potential synergistic effects when combined with BM-MSCs should be investigated in future studies. BM-MSCs have extensively documented strong regenerative abilities, which may support the antifibrotic and immunomodulatory properties of UC-MSCs. Additional studies on cell persistence and potential off-target effects are also needed to evaluate the long-term safety and effectiveness of these therapies in patients with IPF.

From a translational perspective, mesenchymal stem cell–based therapies have been actively explored for pulmonary diseases, including idiopathic pulmonary fibrosis, with an overall acceptable safety profile reported in early clinical studies. Both umbilical cord–derived and bone marrow–derived MSCs have been administered predominantly via intravenous infusion because of its technical feasibility and systemic immunomodulatory effects, while intratracheal or endobronchial delivery has also been investigated to enhance local pulmonary targeting [16], 17], 19], 37], 39]. Although clinical efficacy remains variable and appears to depend on factors such as cell source, dose, timing, and route of administration, the superior antifibrotic and anti-inflammatory effects of UC-MSCs observed in the present study provide experimental support for their potential translational relevance in IPF.

Several limitations of the present study should be noted. Although the bleomycin-induced pulmonary fibrosis model is widely used in experimental research, it does not fully reproduce the complexity and heterogeneity of human idiopathic pulmonary fibrosis. In addition, this study examined a single cell dose and administration protocol, and therefore did not address potential dose–response effects or alternative treatment schedules. Furthermore, the long-term biodistribution, persistence, and *in vivo* fate of the administered mesenchymal stem cells were not evaluated. In addition to these considerations, anti-inflammatory mediators such as IL-10, which have been implicated in the immunomodulatory effects of mesenchymal stem cells in fibrotic lung diseases, were not assessed in the present study. Taken together, these limitations indicate that the present findings should be interpreted with caution, and that further investigations using additional preclinical models and clinical studies will be necessary to clarify the translational relevance of these observations.

Several limitations of the present study should be noted. Although the bleomycin-induced pulmonary fibrosis model is widely used in experimental research, it does not fully reproduce the complexity and heterogeneity of human idiopathic pulmonary fibrosis. In addition, this study examined a single cell dose and administration protocol, and therefore did not address potential dose–response effects or alternative treatment schedules. Furthermore, the long-term biodistribution, persistence, and *in vivo* fate of the administered mesenchymal stem cells were not evaluated. Taken together, these limitations indicate that the present findings should be interpreted with caution, and that further investigations using additional preclinical models and clinical studies will be necessary to clarify the translational relevance of these observations.

## 5 Conclusions

The present study demonstrated that the pathological and fatal features of IPF induced by bleomycin can successfully be replicated. We documented several significant outcomes after administering bleomycin to mice for 14 days, followed by the administration of UC-MSCs and the harvesting of lung tissue on day 42. The administration of UC-MSCs notably decreased mortality rates, body weight loss, and various histopathological alterations. Additionally, we observed reductions in fibrotic lesions and collagen synthesis linked to TGF-*β*, *α*-SMA, and hydroxyproline expression. We also observed a decrease in lung edema and leukocytic infiltration, as indicated by alveolar barrier dysfunction and proinflammatory cytokines. Our findings indicated that in contrast to the administration of BM-MSCs, use of UC-MSCs significantly reduced mortality rates, histopathological alterations, and fibrotic lesions, as evidenced by TGF-*β* and *α*-SMA expression. Administration of BM-MSCs also improved lung edema and reduced leukocytic infiltration, which were associated with alveolar barrier dysfunction and TNFα expression. Based on these findings, we suggest that UC-MSCs exhibit more beneficial effects than BM-MSCs in bleomycin-induced IPF.
